# Multiple Parallel Grafts for Urgent Endovascular Repair of a Ruptured Mycotic Aortic Aneurysm

**DOI:** 10.1055/s-0042-1743200

**Published:** 2022-08-07

**Authors:** Andrea Xodo, Michele Piazza, Jacopo Taglialavoro, Marco Zavatta, Franco Grego, Michele Antonello

**Affiliations:** 1 Division of Vascular and Endovascular Surgery, Department of Cardiac, Thoracic Vascular Sciences and Public Health, Padua University, Padua, Italy

**Keywords:** mycotic aneurysms, parallel grafts, endoleak, coils, thoracic endografts, urgent repair, aortic rupture

## Abstract

A 73-year-old woman underwent urgent endovascular repair of a ruptured mycotic aortic aneurysm. A thoracic stent graft was employed as the main endograft, while the celiac trunk and superior mesenteric artery were revascularized by the chimney technique and the renal arteries through the periscope technique. Postoperative computed tomography revealed a Type A1 gutter, treated by detachable coils and peripheral occlusion devices. Six-month follow-up revealed patency of the stent grafts, without endoleak or stent graft infection signs.

## Introduction

Mycotic aortic aneurysms (MAAs) are rare and life-threatening conditions, with challenging treatment and poor prognosis. 1 We present a case of a ruptured MAA, treated using multiple parallel grafts (PGs) combined with a thoracic endograft deployment.

## Case Presentation

A 73-year-old female was urgently admitted to our clinic from an outside hospital, where she was treated for 2 weeks for evening fever and abdominal pain. Her medical history included arterial hypertension, thrombocytopenia, and a previous midline laparotomy for gastrointestinal stromal tumor (GIST) excision, complicated with acute pancreatitis.

At the admission time, her hemoglobin was 10.2 g/dL, leukocyte count was 9.78 × 10 ^9^ /L, and erythrocyte sedimentation rate was 89 mm/h, while the C-reactive protein level was 61 mg/L. During the first hospitalization, several tests had been performed with negative results (blood culture, human immunodeficiency virus, Brucella, Salmonella, Bartonella, Treponema pallidum, and Quantiferon). Abdominal computed tomography angiography (CTA) revealed a thickening of the aortic wall, with left pleural effusion and periaortic lymphadenopathy. The patient was therefore initially treated with empiric antimicrobial therapy (piperacillin/tazobactan) and corticosteroids.

**Fig. 1:**
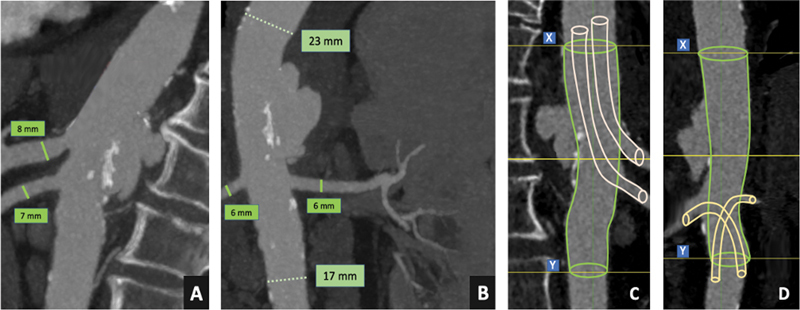
Visceral vessels and aortic diameters ( **A** , **B** ). Preoperative planning: “chimney” technique to ensure the celiac artery and the superior mesenteric artery revascularization, with “periscope” technique for both renal arteries revascularization; *XY* length was 10 cm ( **C** , **D** ).

Despite this therapy, 2 weeks later she developed acute back pain, with initial hemodynamic instability. CTA demonstrated an impeding rupture of the posterior wall of the thoracoabdominal aorta. The rupture originated 20 mm above the origin of the superior mesenteric artery (SMA), while only 7 mm were included between the end of the rupture and the left renal artery, making a suprarenal distal sealing zone inadequate ( Fig. 1A, B ).

Treatment planning initially included open surgery through a left thoraco-phreno-laparotomy or a complete retroperitoneal approach by left pararectal abdominal incision extended to the 9th–11th intercostal space, but these possibilities were discarded due to patient's severe medical history, in particular considering the previous GIST excision and the thrombocytopenia.

Hybrid treatment with median laparotomy, visceral debranching and straight aortic endograft deployment was also hypothesized as an alternative; however, it was excluded due to the previous history of GIST excision complicated by acute pancreatitis.

An off-the-shelf endograft (T-Branch stent graft; Cook Medical, Brisbane, Queensland, Australia) was available, but the diameter of the aorta at the level of the visceral arteries was too small (23 mm) for using it. 2 The use of a custom-made (CM) device was obviously excluded in this urgent setting. An alternative option was a physician-modified fenestrated stent graft, but its use raised concerns about the feasibility and the long-term durability.

**Fig. 2:**
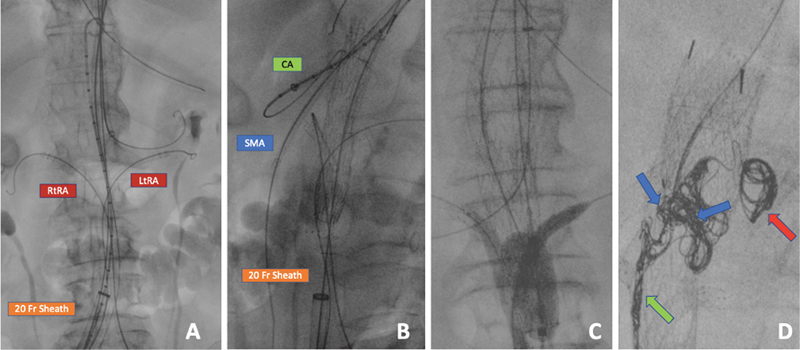
Cannulation of the aortic branch vessels, with a 20-Fr sheath into the right common iliac artery ( **A** ). Both renal arteries were cannulated from the femoral accesses, while celiac artery (CA) and superior mesenteric artery (SMA) cannulations were achieved from the brachial accesses ( **A** , **B** ). Proximal and distal molding into the main endograft by an aortic compliant balloon, respectively, simultaneously with CA/SMA and renal intrastent ballooning ( **C** ). Red arrow shows Ruby detachable coils (32–60 mm) filling a periendograft large cavity. Green arrow indicates peripheral occlusion device (POD) (6 × 50–4 × 30 mm), which are characterized by presence of an anchor segment to fix the system in a high-flow space as the gutter, achieving a high packing density. Blue arrows show POD packing coils, a vascular closure system with an elongated shape designed to fill the spaces left empty by the POD system ( **D** ).

For these reasons, a total endovascular solution through chimney and periscope techniques was planned ( Fig. 1C, D ). The following listed are the surgical steps that were performed:

- Under general anesthesia, bilateral brachial surgical accesses were obtained, as well as right groin access, facilitating double puncture of the femoral artery. A left femoral percutaneous approach was obtained with a retrograde ultrasound puncture.
- The renal arteries were sequentially catheterized from the femoral accesses ( Fig. 2A ), while the celiac artery (CA) and the SMA from the brachial accesses ( Fig. 2B ).
- A TGM 262110E Gore TAG Conformable thoracic endoprosthesis (W. L. Gore & Associates, Flagstaff, AZ) was introduced through the right femoral access and positioned 2 cm over the CA origin.
- 6 × 60 mm Covera Plus stents (Bard, Tempe, AZ) were deployed into the renal arteries under road mapping, 2 cm into the target vessel, taking care to ensure at least 1 cm of the periscope stent below the main graft distal edge.
- Gore TAG graft was therefore unsheathed and partially deployed.
- Through the brachial accesses, 8 × 100 mm and 7 × 100 mm Covera Plus stents were respectively deployed in the CA and in the SMA. A second 8 × 80 mm Covera Plus stent was used to extend the chimney for the SMA, due to the length of the vessel.
- The thoracic endograft was then finally deployed.
- All the covered stents were reinforced with a nitinol uncovered self-expanding stent (Abbott Absolute Pro; Abbott Vascular, Chicago, IL).
- Gore aortic cuff (PLA 320400) was deployed as planned 2 cm above the proximal edge of the main endograft to extend the aortic main component superiorly.
- The proximal and the distal “molding” into the main endograft were performed by an aortic compliant balloon, respectively, simultaneously with CA/SMA and renal intrastent ballooning ( Fig. 2C ).

The final angiogram showed a Type A1 gutter-related endoleak, confirmed at the CTA. 3 The total surgical procedure required 270 minutes and the patient was admitted to the intensive care unit for the postoperative monitoring (2 days).

The patient's clinical condition improved after the procedure and 2 weeks later a second step was planned to fix the endoleak. Left brachial artery surgical reexposure was performed and a 90-cm 7 Fr catheter inserted. Ruby Coils, peripheral occlusion device (POD), and POD packing coils (Penumbra Inc., Alameda, CA) were employed to embolize the gutter ( Fig. 2D ); the procedure time was 140 minutes.

**Fig. 3:**
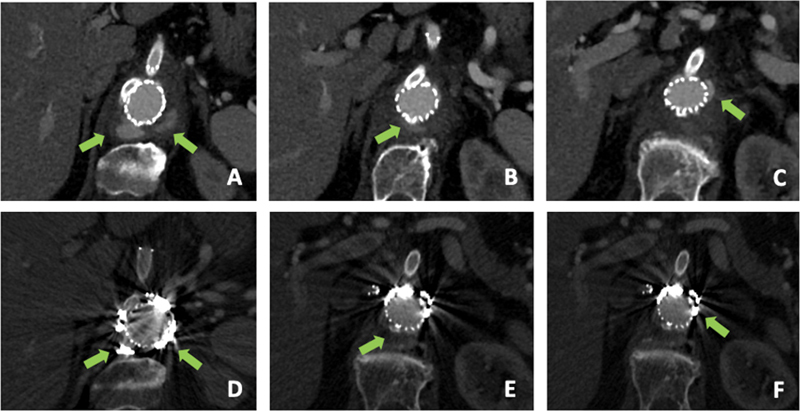
Arrows show the contrast enhancement outside the endograft at the computed tomography angiography (CTA) after the initial repair ( **A** – **C** ). Arrows indicate a good result, without signs of endoleak, in the control CTA 6 months after the procedure ( **D** – **F** ).

The patient was discharged on the 25th postoperative day with no local or general complications. No pathogens were detected by conventional blood culture methods. Intravenous antibiotic therapy was used for the entire period of hospitalization (meropenem 1 g three times daily + vancomycin 500 mg four times daily) and antibiotic treatment with home oral trimethoprim-sulfamethoxazole and amoxicillin/clavulanic acid (respectively 960 mg two times daily + 1 g three times daily) was prescribed by the infectious disease specialist. Control CTA 6 months later showed a complete endoleak resolution, with a regular patency of the stents and without *signs of* stent graft infection ( Fig. 3 ). The patient remained asymptomatic, without any signs or symptoms of infection.

## Discussion

MAAs are rare, with an incidence of approximately 0.6 to 2% of all aortic aneurysms. 4 Surgical approach generally entails radical removal of the infected arterial segment and its surrounding tissues, followed by in situ replacement or biological reconstruction. The management of this disease remains a challenging problem and the surgical outcomes depend on many factors, such as the type of aneurysm, its anatomical location, and the type of surgical treatment; patients with MAA also usually suffer from a compromised general clinical condition, with a profound state of immunosuppression.

Dubois et al 5 reported their experience with 43 patients, affected by mycotic abdominal aortic aneurysms (AAAs), treated by surgical in situ and/or extra-anatomic reconstruction (21 of them presented with ruptured AAA).

In-hospital mortality was 22.7% and 8% of survived patients presented with reinfection during the follow-up. 5

A hybrid technique, by endovascular aortic repair and visceral vessel debranching, avoids the aortic clamping and its possible ischemic complications, as well as the thoracotomy and the use of extracorporeal circulation in case of thoracoabdominal aortic aneurysm repair. Endovascular procedures with CM endografts are generally not available for treatment of patients requiring urgent intervention, while off-the-shelf devices have very specific anatomic inclusion criteria for clinical applicability. Kitagawa et al 6 evaluated aortic anatomy in 66 patients treated by multibranched stent grafts and 12% did not meet all of the anatomical criteria suggested for implantation.

Chimney and periscope techniques, described by Greenberg as “bailout procedure” in case of accidental coverage of an aortic branch, allow the extension of the proximal and distal landing zones preserving the visceral vessels and using devices stocked in most of vascular and endovascular centers. 7 PG has also been shown to be feasible in cases of patients with juxtarenal AAA, considered unfit for fenestrated endovascular aortic repair (FEVAR) for clinical, anatomical, technical, or endograft “manufacturing time reasons.” 8

Lachat et al, 9 in 2010, reported a case of ruptured thoracoabdominal aortic aneurysms treated with a quadruple chimney-periscope technique; however, in this case the pathology was not of mycotic etiology.

Preoperative risk stratification should always be performed, in particular for these challenging scenarios; surgical risk scores are simple tools to use to calculate the average surgical risk and can help evaluate the most appropriate operative strategy; however, they do not accurately predict results when one or more serious complications occur. Furthermore, in the case of MAA, both the open and the endovascular procedure require a high level of experience and knowledge of the pathology on the part of the surgeons, which should guide the choice of the type of intervention.

A recent systematic study concerning the management of MAAs, published by Sörelius et al, 10 showed that MAAs are complicated by infection-related complications in 21%, irrespective of surgical technique. The choice of materials is a crucial issue for *CHIM* ney, *P* eriscope, and *S* norkel ( *CHIMPS* ) technique: self-expanding stents, such as Covera Plus, offer high conformability, associated with a low profile sheath, but apply only a reduced radial force compared with balloon-expandable stents, which are more rigid but guarantee a high radial force with great precision during the release. Additionally, for this type of stent, deployment and molding occur in a single step.

Some technical aspects of this case deserve consideration. The Gore TAG Conformable Thoracic endograft with active control system has facilitated a precise and “staged” deployment of the stents; in fact, this allows partial opening of the thoracic endoprosthesis to 50% of its diameter, from the proximal to the distal end, followed by the final deployment from the distal to the proximal edge. Uncovered bare metal stents have been used for all vessels to reinforce the area apparently more subject to compression by the main endograft. The high number of periscope grafts might increase the risk of gutter issues, as stated by Tanoius et al. 11 However, we have chosen the configuration with two chimney and two periscope grafts, trying to reduce this risk.

How to size the main aortic endograft using CHIMPS is still an open issue: as known, the internal circumference of the aorta significantly increases in the case of one or two PGs.

Considering the percentage of increase in the internal circumference, in our clinical practice we usually chose an endograft with an added oversize value (AOV) of 25% in case of a single PG and of 45% in case of two PGs; the AOV is calculated on the standard oversizing (SO) that we usually consider for standard endovascular aneurysm repair/thoracic endovascular aortic repair, in the absence of periscope or chimney/snorkel technique and according to aortic diameter and different type of pathology to treat (aneurysmatic disease, dissection, rupture, etc.).

In this case, due to the disease and the mismatch between proximal and distal landing zones (23 vs. 17 mm), we considered a 10% of SO, to reduce the risk of endograft collapse below the renal arteries. The final oversizing (FO) was therefore (10% + (45%*10%)) = 14.5%.

Considering this simple calculation and the aortic diameter (23 mm at the proximal landing zone), we had therefore chosen a 26-mm main endograft (FO = 14%).

Penumbra Ruby coils, POD, and POD packing coils were employed to treat a Type A1 gutter-related endoleak. Detachable coils help to reduce the risk of distal embolization and the availability of different lengths with a high packing density seems suitable to occlude large vessels or a large endoleak. One of the best features is the softness, which facilitates the embolization, even with a relative instability of the catheter, reaching a very distal site and achieving good packing in a narrow space. The POD is instead designed specifically to pack the vessels very densely; in this case, after coil deployment, POD was successfully deployed to create a dense mechanical occlusion in a short, high-flow segment.

In summary, in this case, multiple PGs with a quadruple chimney/periscope configuration, combined with thoracic endograft deployment, allowed a complete renovisceral revascularization as an alternative to open surgery (or to complex procedures such as branched endovascular aneurysm repair and FEVAR) for the urgent treatment of a ruptured MAA. Embolization devices such as large volume detachable coils and PODs seem effective to conduct an efficient embolization in case of gutter-related endoleak, in particular for dense and precise vessel packing.

The crucial question of durability of endovascular treatment in case of MAAs remains still debated: for this reason, long-term durability will need to be evaluated, assessing for possible late infection-related complications.
